# The height-, weight- and BMI-for-age of preschool children from Nizhny Novgorod city, Russia, relative to the international growth references

**DOI:** 10.1186/s12889-016-2946-8

**Published:** 2016-03-17

**Authors:** Ekaterina Nazarova, Yury Kuzmichev

**Affiliations:** Privolzhsky Federal Medical Research Centre, Nizhny, Novgorod Russia; Nizhny Novgorod State Medical Academy, Nizhny, Novgorod Russia

**Keywords:** Preschool children, Growth charts, Growth references, Height, Weight, Body mass index, Height-for-age z-score, Weight-for –age z-score, BMI z-score

## Abstract

**Background:**

Monitoring a child’s growth status helps to diagnose diseases and implement curative and preventive measures.

The aim of this study was to assess how well preschool children of Russian city (Nizhny Novgorod) match with, or diverge from, international growth charts (WHO2006,2007; USCDC2000).

**Methods:**

Cross-sectional study included 3,130 children aged 3–7 years attending municipal preschools of Nizhny Novgorod, the city in the European part of Russia. The study was held from February 2012 to October 2013.

The international WHO2006,2007 and USCDC2000 growth references were used to calculate the height, weight and BMI z-scores. The distributions of z-scores were analysed with descriptive and inferential statistical methods. Z-score equal 0.25 was considered as a benchmark for clinically significant differences.

**Results:**

Means height z-scores calculated with the use of WHO2006, 2007 and USCDC2000 references were above the 50th centile (0.13 – 0.47) for both boys and girls. The means height z-scores was less than 0.25 SD above the 50th centile only for WHO2006.

Stunting prevalence (the height-for-age z-score less than -2) was slightly higher under WHO2006 (3-4 %) than under USCDC 2000 (2–3 %). Stunting prevalence among children aged 5–7 years was similar under WHO2007 and USCDC2000 references (1 %).

For boys and girls aged 3–4 years the thinness prevalence, using WHO2006 was 2 %, using USCDC2000 was 6 % (*p* < 0.05). At the age 5–7 years this proportion under WHO2007 was 3 % in both sex groups, under USCDC2000 was 8 % for boys and 6 % for girls (*p* < 0.05).

A proportion of preschoolers aged 3–4 years with overweight was slightly higher under WHO2006 reference (13–15 %) than under USCDC2000 (12–14 %). In the case of age 5–7 years the overweight prevalence under WHO2007 (13–12 %) was lower than under USCDC2000 (14 %).

Obesity prevalence under WHO2006,2007 (3–4 %) was slightly higher than that under USCDC2000 reference (2–3 %).

Preschoolers’ distribution by groups of normal weight, overweight, obesity didn’t significantly differ among the references (chi-square).

**Conclusions:**

The growth assessment of children aged 3–7 years attending municipal preschools of the Russian city Nizhny Novgorod under the international references (WHO2006,2007; USCDC 2000), demonstrated that the height fit to the WHO2006 standard for the children aged 3 and 4 was generally fine, since all the mean values were within 0.25 of the standard deviations of the mean. Beyond the age of 5 the fit to the WHO2007 was poor while the fit to the USCDC2000 was poor throughout.

**Electronic supplementary material:**

The online version of this article (doi:10.1186/s12889-016-2946-8) contains supplementary material, which is available to authorized users.

## Background

Growth is one of the most important indicator of children health. Height reflects the long term malnutrition, weight shows short term problems. Nutrition disorders can be particularly serious in children, since they interfere with growth and development, and may predispose to many health problems, such as infection and chronic disease. Stunting, or being too short for one’s age, has long-term effects on individuals and societies, including: diminished cognitive and physical development, reduced productive capacity and poor health, and an increased risk of degenerative diseases [1]. Overweight and obesity is a major risk factor for diseases such as cardiovascular disease, type 2 diabetes and many cancers (including, colorectal cancer, kidney cancer and oesophageal cancer). These diseases, often referred to as noncommunicable diseases, not only cause premature mortality but also long-term morbidity. In addition, overweight and obesity in children are associated with significant reductions in quality of life [2].

Height and weight monitoring helps to duly diagnose diseases and implement curative and preventive measures.

There is no national growth reference in Russia due to a diversity of climate and geographical areas, nationalities and ethnic groups and a difference of social and economic situation in the country regions [3]. Regional growth charts were worked out in some Russian regions. The Nizhny Novgorod regional growth chart for preschool children was approved in 2004.

In 2000 US CDC released growth charts for children from birth to 20 years. The CDC growth curves are based on children living in the United States.

In 2006 the WHO published standards for assessing the growth of children from birth to 5 years of age based on a multi-country study (Brazil, Ghana, India, Norway, Oman and USA) on growth of healthy breast-fed children under optimal conditions. The charts were derived from growth data of children, who participated in the WHO-Multicenter Growth Reference Study between 1997 and 2003.

In 2007, WHO reference for children from 5 to 19 years was released. It is based on the growth of American children, on re-analysis of National Centre for Health Statistics data from 1977.

A systematic review describing papers that have tested international standards found that generally the means for height z-score calculated under WHO 2006 were no more than 0.5SD above or below the 50th centile. Among 0.5SD outliers at three or more ages, Europeans (Netherlands, Finland) were generally above 0.5 SD and some other groups (eg, Saudi Arabians and Asian Indians) were below −0.5 SD. Thus, the curves may underindicate short stature in slightly taller European populations and overindicate it in shorter ones. Clinicians should keep this fact in mind when dealing with children from these populations [4].

UK children match the WHO2006 standards well for length and height at all ages and for weight in the early weeks. At age 12 months, the risk of being classified as underweight (weight <2nd centile) was considerably lower according to the WHO standard than by the UK 1990 Growth Reference, and the risk of being classified as obese at 4–5 years (body mass index >98th centile) was slightly increased [5, 6].

Growth data from French children, born between 1981 and 2007, from studies in general populations, showed that their growth was closer to the WHO2006, 2007 growth charts than to the current French references, except from birth to 6 months of age [7]. But other authors found considerable differences in mean height among European populations, with children from Northern Europe generally being taller then those from Southern Europe, and advocated using recent national or European height-for-age charts derived from recent national data [8].

There are no epidemiologic data in medical publications concerning the information value of implementing international references in Russia. The aim of the present analysis was to assess how well preschool children of the Russian city match with, or diverge from international growth charts.

## Methods

Nizhny Novgorod is a major industrial city in the European part of the Russian Federation with a population of 1,263,000 people. As of 01.01.2014, municipal preschools were attended by 49640 children of 3–7 years.

Cross-sectional study of anthropometric indices for 3–7 year-old children was held from February 2012 to October 2013. The research was performed with the approval of Department of Education of the Municipal Administration and Research Institute of Pediatric Gastroenterology local ethics committee (21–02.04.10). The study was carried out in 24 municipal preschool institutions chosen by random sampling from all municipal city districts. The randomization was performed via the table of random numbers generated in Statistica. The study included all the children who were present at the institutions during the study days and which parents signed Informed Consent. Parents of 15 preschoolers refused to participate in the study – these children were excluded. So the study included 3,130 preschoolers (1,625 boys and 1,505 girls) attending selected institutions (95 % from all preschoolers in the selected kindergartens).

Body height and weight were measured in the morning hours by trained medical officers. Height was measured twice by the same person (in case of a difference between measurements exceeding 4 mm, a third measurement was taken) using Тanita HR-001 height meter in the standing position (with no shoes), to the nearest millimeter. Body weight was measured in light underwear to the nearest 0.05 kg, using a digital medical scale Тanita BC-418 МА. Body mass index (BMI) was calculated as body weight (kg) divided by height in meters squared.

Individual age was calculated in months as of the date of the examination.

Anthropometric data of preschool children was compared to WHO2006 [9], WHO2007 [10, 11], USCDC2000 references [12].

These references were used to calculate height, weight and BMI z-scores.

Z-scores relative to the WHO2006,2007 references were calculated with the SAS code provided by the WHO Anthro Team.

Z-scores relative to the USCDC2000 were calculated with the SAS code downloaded from http://www.cdc.gov/nccdphp/dnpa/growthcharts/resources/sas.htm.

The means and 95 % CI of height, weight and BMI z-scores were calculated separately for each sex and for each age chart. Differences from zero of the means of z-scores were analyzed with Student’s *t*-test for the whole age range (3 to 7 years of age) and separately for each year of age. The WHO used 0.5 as z-score benchmark for clinically significant differences [13, 14]. We adopted this cut-off. A mean difference that was less than 0.25 of a standard deviation was considered very unlikely to have any important clinical implication. Differences of z-scores means between reference ranges were analyzed with the paired *t*-test. A child was considered stunted (low height-for-age) if the height-for-age z-score was below −2. Percentage of stunted children were calculated. Differences in the distribution of stunting according to sex and reference range were tested with the McNemar test. Differences in the prevalence of stunting between sex were tested with the chi square test. Differences in the distribution of thinness, normal weight, overweight and obesity according to sex and reference range were tested with the chi square test. A variation in the proportion of children was considered to be important unless it exceeds more than one percentage points. Data were processed with the MsAccess database and MsExcel spreadsheet. All analyses were conducted with SAS 9.1 for Windows.

## Results

Additional file 1: Table S1. Height, weight and BMI by sex and age of Nizhny Novgorod preschoolers included in the analysis.

Table 1 shows the means of height z-scores, calculated using WHO2006,2007, USCDC2000 references. All averages were positive. The confidence intervals of means for all references did not include zero. Means height z-scores were less than 0.25 only for WHO2006. Means height z-scores calculated under WHO2006 and USCDC2000 references significantly differed from each other. Statistically differ of means between WHO2007 and USCDC2000 was not observed.

**Table 1:** Russian preschool children mean height, weight and BMI z-scores relative to the three references

Reference	N	Height z-score			Weight z-score			BMI z-score		
		Mean	95 % CI		Mean	95 % CI		Mean	95 % CI	
boys aged 3–4 years										
WHO2006	643	0.13	0.04	0.20	0.17	0.10	0.25	0.13	0.10	0.21
USCDC2000		0.28*	0.22	0.37	0.15	0.07	0.24	−0.19	−0.29	−0.1
girls aged 3–4 years										
WHO2006	599	0.15	0.07	0.24	0.17	0.09	0.24	0.09	0.01	0.17
USCDC2000		0.46*	0.37	0.54	0.18	0.10	0.26	−0.08	−0.17	0.02
boys aged 5–7 years										
WHO2007	982	0.34*	0.28	0.42	0.23	0.16	0.29	−0.01	−0.08	0.06
USCDC2000		0.38	0.31	0.44	0.12	0.05	0.18	−0.2	−0.28	−0.11
girls aged 5–7 years										
WHO2007	906	0.44*	0.37	0.51	0.24	0.18	0.31	−0.01	−0.07	0.06
USCDC2000		0.47	0.40	0.54	0.18	0.11	0.25	−0.07	−0.15	0.01

* - *p* < 0.05 between sex groups

In sex analysis, statistically significant difference of height z-score between boys and girls was registered for children aged 3–4 years (USCDC2000) and 5–7 years (WHO2007).

**Fig. 1:**
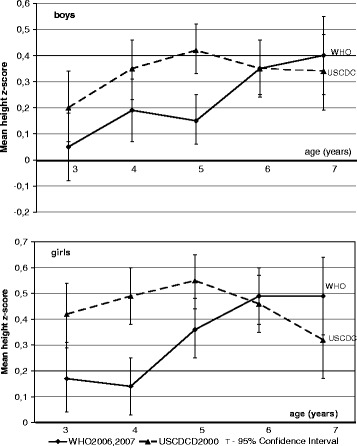
Russian preschool children means of height z-score relative to three growth charts. Shows the means of height z-score calculated using three references plotted against age for boys and girls separately

In age analysis, the mean height z-score was positive over the whole age range (both boys and girls) (Fig. 1). The lower CI was slightly negative 0 (−0.08) only for boys aged 3 under WHO2006. Means height z-score were less 0.25 only for boys and girls aged 3 and 4 years calculated under WHO2006.

Table 2 shows stunting prevalence calculated using WHO2006,2007, USCDC2000 references. Stunting prevalence among the boys under 5 years was at the level of 4.35 % (WHO2006) and 3.27 % (USCDC2000). Stunting prevalence among the girls under 5 years was 2.84 % (WHO2006) and 1.84 % (USCDC2000). We have not revealed a statistically significant difference in stunting prevalence between the references. No significant difference of this index has been detected between sex groups.

**Table 2:** Stunting prevalence and BMI distribution among Russian preschool children using the three reference

	N	WHO n %	USCDC n %	95%CI WHO		95%CI USCDC	
Stunting (height-for-age z-score < −2)							
boys aged 3–4 years	643	28	21				
		4.35	3.27	2.91	6.23	2.03	4.95
girls aged 3–4 years	599	17	11				
		2.84	1.84	1.66	4.51	0.92	3.26
boys aged 5–7 years	982	14	13				
		1.43	1.32	0.78	2.38	0.71	2.25
girls aged 5–7 years	906	16	15				
		1.77	1.66	1.01	2.85	0.93	2.72
Thinness (BMI z-score < −2)							
boys aged 3–4 years	643	13	42				
		2.02	6.53	1.08	3.43	4.75	8.73
girls aged 3–4 years	599	12	35				
		‘2.02	5.84	1.04	3.47	4.10	8.03
boys aged 5–7 years	982	34	76				
		3.46	7,74	2.41	4.80	6.15	9.59
girls aged 5–7 years	906	24	50				
		2,65	5.52	1.70	3.92	4.12	7.21
Normal weight (−2 ≤ BMI z-score ≤ 1)							
boys aged 3–4 years	643	516	504				
		80.25	78.38	76.93	83.26	75.00	81.51
girls aged 3–4 years	599	478	461				
		79.8	76.96	76.36	82.94	73.38	80.28
boys aged 5–7 years	982	781	739				
		79.35	’79.25	76.87	82.01	72.43	77.93
girls aged 5–7 years	906	740	704				
		81.68	77.70	79.0	84.15	74.85	80.38
Overweight (1 < BMI z-score ≤ 2)							
boys aged 3–4 years	643	84	74				
		13.06	11.51	10.56	15.92	9.15	14.23
girls aged 3–4 years	599	88	85				
		14.69	14.19	11.95	17.78	11.49	17.24
boys aged 5–7 years	982	124	134				
		12.63	13.65	10.61	14.87	11.56	15.95
girls aged 5–7 years	906	110	130				
		12.14	14.35	10.09	14.45	12.13	16.80
Obesity (BMI z-score > 2)							
boys aged 3–4 years	643	30	23				
		4.67	3.58	3.17	6.59	2.28	5.32
girls aged 3–4 years	599	21	18				
		3.51	3.01	2.18	5.31	1.79	4.71
boys aged 5–7 years	982	46	33				
		4.38	3.36	3.45	6.20	2.32	4.69
girls aged 5–7 years	906	32	22				
		3.53	2.43	2.43	4.95	1.53	3.65

Stunting prevalence among children aged 5–7 was similar under WHO2007 and USCDC2000 references. It was 1.43 and 1.32 % for boys respectively, and 1.77 and 1.66 % for girls.

As we see at the Table 1, means weight z-score calculated under WHO2006,2007 and USCDC2000 were positive and less 0.25 both for boys and girls. 95 % CI didn’t include zero.

**Fig. 2:**
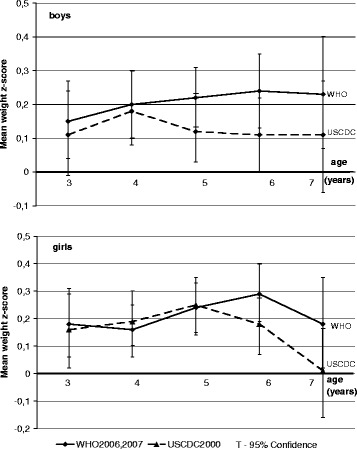
Russian preschool children mean weight z-scores calculated using three weight-for-age references. Shows the mean weight z-score calculated using three weight references plotted against age for boys and girls separately

Figure 2 shows the mean weight z-score calculated using three weight references plotted against age for boys and girls separately. Means weight z-score calculated under international references were positive irrespective of the age and sex. Mean weight z-score was above 0.25 only for girls aged 6 under WHO2007. There was not statistically significant difference between standards both for boy’s and girl’s groups.

As we see at the Table 1, all means BMI z-score were less than 0.25 as to WHO2006, 2007 and USCDC2000 growth charts. Mean BMI z-score for boys aged 3–4 years was above 0 (0.13) using WHO2006 reference and below 0 (−0.19) using USCDC2000 reference. As for boys aged 5–7 years mean BMI z-score calculated under WHO2007 is сlose to 0 (−0.01) and calculated under USCDC2000 is negative (−0.2). The differ between the references was statistically significant (*p* = 0.000). As for girls, at the age under 5 years mean BMI z-score сalculated using WHO2006 is slightly above zero (0.09) and calculated using USCDC2000 is slightly below zero (−0.08). Means BMI z-score calculated for girls aged 5–7 years under WHO 2007 and USCDC2000 are below zero (−0.01 and −0.07) (Table 1). There was not statistically significant difference between the charts for girls.

**Fig. 3:**
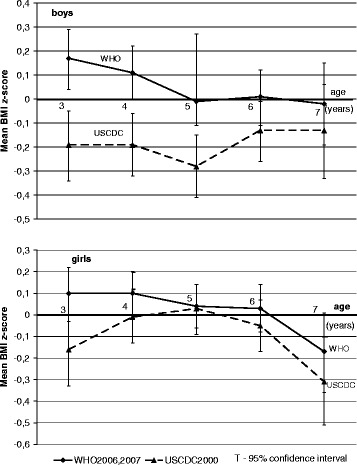
Russian preschool children mean BMI-z-scores calculated using three BMI-for-age references. Shows the mean BMI-z-scores calculated using three references plotted against age for boys and girls separately

Figure 3 shows that means BMI z-score were less −0.25 only for boys aged 5 and girls aged 7 under USCDC2000. Means BMI z-score calculated using WHO2006 for boys 3–4 aged were above 0, close to zero for 5–7 years boys (WHO2007). Means BMI z-score calculated using USCDC2000 were negative for boys of all age groups. There were not significant differences between references for boys aged 6 and 7 years. In the case of girls, the mean BMI z-score calculated with the use of WHO reference was negative only for the girls aged 7 years (−0.17). Means BMI z-score calculated under USCDC2000 reference were negative in girls aged 3 (−0.16), 6 (−0.05), 7 (−0.31), were close to zero in girls at the age 4–5 (Fig. 3). There were not statistically significant difference between references in girls all age range.

Table 2 demonstrates Russian preschool children BMI distribution. The proportion with thinness (ВMI z-score < −2) calculating under WHO references was less by half than using USCDC2000 in both sex groups (*p* < 0.05). For boys and girls aged 3–4 years the thinness prevalence, using WHO2006 was 2.02 %, using USCDC2000 was 6.53–5.84 %. At the age 5–7 years the thinness prevalence under WHO2007 was 3.46 % for boys and 2.65 % for girls, under USCDC2000 7.74–5.52 % respectively.

A proportion of boys aged 3–4 years with overweight was slightly higher under WHO2006 reference (13.06 %) than under USCDC2000 growth chart (11.51 %). For girls the overweight prevalence was similar under WHO2006 (14.69 %) and USCDC2000 (14.19 %). In the case of age 5–7 years the overweight prevalence under WHO2007 was lower than under USCDC2000. For boys this proportion was 12.63 % (WHO2007) and 13.65 % (USCDC2000), for girls this proportion was 12.14 % (WHO2007) и 14.35 %(USCDC2000).

Obesity prevalence (BMI z-score >2) among preschoolers сalculated under WHO2006,2007 references was slightly higher than the same one calculated under USCDC2000 reference in both age groups. For boys aged 3–4 years these figures were 4.67 %(WHO2006) и 3.58 % (USCDC2000), for girls of this age group these levels were 3.51 % (WHO2006) и 3.01 % (USCDC2000). For preschoolers aged 5–7 years obesity share for boys was 4.38 % (WHO2007) и 3.36 % (USCDC2000), for girls was 3.53 % (WHO2007) and 2.43 %(USCDC2000).

Russian preschoolers’ distribution by normal weight, overweight and obesity groups under WHO2006,2007 and USCDC2000 references didn’t statistically differ (chi-square).

## Discussion and conclusions

Means height z-score of all ages preschool children in the Russian city using WHO2006,2007 and USCDC2000 standards had the same shift direction - to the right of zero. Means height z-scores were less than 0.25 only for WHO2006. Means height z-score significantly differed calculated under WHO2006 and USCDC2000, didn’t differ under WHO2007 and USCDC2000.

WHO2006 standard is the outcome of multi-country study. USCDC2000 and WHO2007 references describe the growth of US children, and do not represent an international sample. USCDC2000 and WHO2007 growth charts are mainly based on data collected more than forty years ago. Our previous study of Russian preschoolers’ growth dynamics for the last 40 years revealed that modern preschool children are characterized by other total body proportions compared to the children of the late 60s and the early 80s [15].

Thus, the growth assessment of Russian children aged 3–7 years under the three references (WHO2006,2007; USCDC 2000) demonstrated the expected result: the height fit to the WHO2006 standard for the children aged 3 and 4 was generally fine, since all the mean values were within 0.25 of the standard deviations of the mean. Beyond the age of 5 the fit to the WHO2007 was poor while the fit to the USCDC2000 was poor throughout.

Stunting prevalence calculated under WHO2006 reference was slightly higher than USCDC2000 reference calculation but without significant difference. It is observed in medical literature that stunting prevalence calculated under WHO2006 references is higher than USCDC2000 reference calculation [16, 17]. Stunting prevalence among children aged 5–7 was similar under WHO2007 and USCDC2000 references.

Weight z-scores of Russian preschool children calculated under WHO2006, 2007 and USCDC2000 were positive and less than 0.25 for all ages.

BMI z-scores calculated using WHO references were less than 0.25 for all ages in range, were positive or close to zero except girls aged 7 years (−0.17). BMI z-scores calculated under USCDC2000 were mostly negative but didn’t descent below “-0.3”.

Thinness prevalence calculated using WHO2006,2007 references (2–3 %) was significantly less than that under USCDC2000 reference (5–7 %).

A proportion of preschoolers with overweight was just higher under WHO2006 reference (13–15 %) than under USCDC2000 (12–14 %). In the case of age 5–7 years the overweight prevalence under WHO2007 (13–12 %) was lower than under USCDC2000 (14 %). A similar level of overweight prevalence is observed among preschoolers in Spain, Italy, USA, Brazil and Iran [18–22].

Obesity prevalence under WHO2006,2007 (3–4 %) was slightly higher than that under USCDC2000 reference (2–3 %). This level is in line with the obesity prevalence of Italian, Austrian and Iranian preschoolers published in medical literature [19, 22, 23].

Our results confirm medical literature data about the lower rates of thinness and higher rates of overweight and obesity when based on the WHO2006 standards vs USCDC2000 [16, 17].

In general, international WHO2006 standard would be preferred for growth assessment of Russian preschool children.

## Additional file

Additional file 1: Table S1. Russian preschool children height, weight and BMI by sex and age**.** Table provides descriptive statistics of height (mean, median, min, max, SD, 95 % CI), weight and BMI (mean, median, min, max, 95 % CI) by sex and age. (XLS 32 kb)

## Abbreviations

BMI: body mass index
CDC: centers for disease control and prevention
CI: confidence interval
SD: standard deviation
WHO: World Health Organization
